# Cash transfers for HIV prevention: considering their potential

**DOI:** 10.7448/IAS.16.1.18615

**Published:** 2013-08-23

**Authors:** Lori Heise, Brian Lutz, Meghna Ranganathan, Charlotte Watts

**Affiliations:** 1 Faculty of Public Health and Policy, Department of Global Health and Development, The London School of Hygiene and Tropical Medicine (LSHTM), London, UK; 2 United Nations Development Programme (UNDP), HIV, Health and Development Practice, Bureau for Development Policy, New York, NY, USA

**Keywords:** cash transfers and HIV, social protection and HIV, structural drivers and HIV, conditional cash transfers (CCTs) and HIV, HIV and incentives

## Abstract

**Introduction:**

Cash payments to vulnerable households and/or individuals have increasingly garnered attention as a means to reduce poverty, improve health and achieve other development-related outcomes. Recent evidence from Malawi and Tanzania suggests that cash transfers can impact HIV-related behaviours and outcomes and, therefore, could serve as an important addition to HIV prevention efforts.

**Discussion:**

This article reviews the current evidence on cash transfers for HIV prevention and suggests unresolved questions for further research. Gaps include (1) understanding more about the mechanisms and pathways through which cash transfers affect HIV-related outcomes; (2) addressing key operational questions, including the potential feasibility and the costs and benefits of different models of transfers and conditionality; and (3) evaluating and enhancing the wider impacts of cash transfers on health and development.

**Conclusions:**

Ongoing and future studies should build on current findings to unpack unresolved questions and to collect additional evidence on the multiple impacts of transfers in different settings. Furthermore, in order to address questions on sustainability, cash transfer programmes need to be integrated with other sectors and programmes that address structural factors such as education and programming to promote gender equality and address HIV.

## Introduction

Cash payments to individuals and households have increasingly garnered attention as a means to reduce poverty and achieve other social goals, such as improved health and education. The trend towards using cash as a health and development strategy first began in Latin America, where transfers became popular in the 1990s as a social protection mechanism and as a vehicle to encourage behaviours such as immunizing children and ensuring they attend school [1–3]. These transfers were often contingent upon compliance with certain conditions, and hence became known as conditional cash transfers (CCTs).

More recently, interest in cash transfers has spread to sub-Saharan Africa, with 37 countries in 2010 hosting either government-sponsored programmes or pilot initiatives sponsored by donors [4]. Unlike in Latin America, most cash transfer programmes in sub-Saharan Africa are unconditioned and are designed to address a range of challenges, including food insecurity, natural disasters, human capital development and the devastating effects of HIV infection on families and vulnerable children [4].

Evaluation research suggests that under certain conditions, cash transfers can significantly increase household consumption, reduce poverty and food insecurity, increase school enrolment and retention and improve health and nutritional outcomes [4,5]. In high-income countries, provision of cash has also been used to motivate specific health-related behaviours, including smoking cessation [6,7], losing weight [8,9], taking medicine [10] and using certain health services [11].

Cash transfers have recently been explored as a potentially important component of a comprehensive HIV response [12,13]. Applications of cash transfers vary from directly rewarding HIV-protective behaviours to addressing some of the structural factors that shape risk behaviours (such as economic and gender inequalities and lack of educational opportunities). In eastern and southern Africa, for example, research trials have assessed the impacts of providing incentives to collect HIV test results [14], maintain HIV-negative status for a year [15], keep adolescent girls and orphans in school [16–18], encourage young men and women to remain free of sexually transmitted infections (STIs) [19] and provide cash support to households of people living with HIV [12].

The purpose of this commentary is to outline the theory that underpins the use of cash transfers, discuss the evidence of cash transfers’ impacts on HIV-related outcomes and discuss gaps for future research.

### Theoretical underpinnings and debates

Two theoretical perspectives underpin the use of cash transfers as a mechanism of social policy. The first focuses broadly on poverty reduction, with conditionality, if applied, being used to encourage behaviours that have social benefits. Here cash payments are intended to improve structural factors, such as the socio-economic situation of vulnerable groups, and to promote social goods, such as school attendance or childhood immunizations [16]. The second, incentive-based perspective builds upon a large body of work developed by psychologists and behavioural economists in the field of “contingency management” (CM) [20]. In this paradigm, cash payments are provided as incentives to “nudge” individuals towards healthy behaviours. While standard economic theory assumes that fully informed individuals make sensible decisions after weighing the pros and cons of different choices [21], developments in behavioural economics have demonstrated incongruities between theoretical models and human rationality. Thus, in some instances, individuals do give more weight to short-term gratification than to the long-term consequences of risky behaviours. The theory behind CM is to bring forward in time the benefits of avoiding high-risk behaviours.

Recent experimentation on using cash transfers for HIV prevention mirrors these two theoretical approaches. One set of studies explores the potential of cash transfers to affect HIV vulnerability indirectly by addressing structural sources of risk (“upstream factors”). In the short term, for example, direct cash payments may improve household living conditions, thereby reducing poverty-related stress and decreasing the likelihood that girls or women will resort to transactional sex to obtain food or other goods [16]. Adding a conditionality – like keeping one's daughter in school – may further reduce HIV risk by catalyzing girls’ aspirations for the future and delaying their sexual debut. Studies on the feasibility and impact of introducing education-related conditions into previously non-conditioned cash transfer programmes are currently underway in South Africa [22].

A second set of experiments, based on the CM theory, links conditionality directly to HIV-related outcomes (such as collecting HIV test results or maintaining HIV status). For HIV, people have to balance the long-term costs of adopting behaviours that decrease their HIV risk with the short-term benefits of not doing so (e.g., more pleasurable sex, or acquiring food or cash through sexual exchange). Although a purely “rational” assessment should encourage people to be cautious and avoid risky sexual encounters, some individuals will underestimate future risk or give strong preference to immediate gratification over longer term, less certain negative consequences [21].

The wisdom and ethics of using cash transfers, especially CCTs, as a public health strategy are hotly debated [23,24]. Some consider direct cash payments more empowering than traditional sources of government or development aid. They argue that cash payments put money directly into the hands of poor people and give them the freedom to decide how best to use it. This situation stands in contrast to conventional social welfare programmes or “development” projects where donors and/or governments determine what is in the best interests of the poor [25]. Others, however, object to “conditionality” as a public health strategy because it can be seen as paternalistic as well as potentially manipulative [2]. Still others are concerned that incentive schemes may breed dependency and undermine voluntary action to pursue health-seeking behaviours in the absence of incentives [2,4].

Conditionalities are not just questions of public health and ethics; they may also serve political purposes. In Latin America, many of the social protection schemes that include cash transfers were sold to a reluctant middle class by emphasizing the health and educational requirements that accompanied the cash transfer. Conditionality helped increase public support by countering notions that transfers are welfare hand-outs that threaten to undermine personal responsibility and initiative [2].

### Evidence on the HIV impact of cash transfers

Pettifor *et al*. [22] recently reviewed the current evidence on the impact of conditional and unconditional cash transfer programmes on HIV. Most were conducted among adolescents. Of the 10 completed studies, the majority assessed whether conditionality impacted HIV-related behaviours, such as collecting HIV test results or reported condom use [14,15,19]; only a few collected evidence on biological outcomes (e.g. STI or HIV infection) [15,16,19,22]. Of the completed evaluations and the six additional studies underway, most address upstream drivers of HIV vulnerability rather than directly incentivize less risky behaviour.

To date, there is no empirical support for offering cash incentives to individuals to remain HIV-negative [15]; the only study conducted so far found no impact of transfers on HIV status or reported sexual behavior. Indeed, making cash benefits contingent on HIV status raises ethical concerns, with the possibility of further stigmatizing and disempowering already vulnerable groups. The withdrawal of payments following HIV diagnosis may also increase economic hardship and serve to communicate unwittingly people's HIV status to their families and communities. Kohler and Thorton [15] tried to minimize this risk by allowing people deemed HIV-positive at baseline to continue with the study, but they did not make similar accommodations for individuals who stopped receiving payments because they became HIV-positive during the study.

The RESPECT study, which linked transfers to curable STIs, however, did demonstrate some success. Beneficiaries were provided cash rewards every 4 months for remaining free of chlamydia, gonorrhoea and syphilis. After 1 year, the study recorded a 25% drop in the incidence of STIs (9% in the intervention vs. 12% in the control group) [95% confidence interval (CI) 0.47–0.99] [19]. By linking cash to curable STIs as a proxy for HIV risk rather than to HIV status *per se*, the study's approach avoided stigmatizing people living with HIV.

Other evaluation studies are attempting to demonstrate that transfers can affect HIV-related behaviours without targeting HIV directly. In the Zomba cash transfer trial in Malawi, for example, adolescent girls and their households were given monthly cash stipends of varying amounts. Some girls received unconditional stipends, while others received stipends conditional on school attendance. Schoolgirls who received monthly cash payments were significantly less likely than girls who did not receive payments to be infected with HIV (1.2% vs. 3.0%; OR 0.36, 95% CI 0.14–0.91) or herpes simplex virus-2 (HSV-2) (0.7% vs. 3.0%; OR 0.24, 0.09–0.69) in the short term [16]. The impact was the same in both the conditioned and unconditioned groups. Subsequent analyses suggest that one way the intervention worked was to reduce the frequency of transactional sex. Girls who received transfer money were less likely to have older sexual partners and had less frequent sex [16].

With a 64% relative reduction in HIV prevalence, the Zomba trial offers some exciting possibilities; nonetheless, it is likely that cash transfers will need to be paired with other structural and HIV-specific interventions to have a sustained, population-level impact on HIV. The Zomba trial, for example, did not increase condom use and did not include activities designed directly to empower girls or to strengthen their HIV-related knowledge and skills [13]. Moreover, in absolute terms, the impact of the intervention on sexual behaviour was small, as only 2.5% of girls in the control group versus 0.5% in the intervention group had older sexual partners [13]. Thus, while the Zomba trial provides proof of concept that anti-poverty efforts can reduce HIV risk, there may also be room to achieve greater or broader impacts by adding additional components to cash transfer programmes.

## Discussion

### So where does this lead us?

These findings represent the start of an important evidence base, but many unanswered questions remain. Further research is needed to help address three broad categories of questions: (1) What are the mechanisms and pathways through which cash transfers affect HIV-related outcomes, (2) what key operational and programmatic questions remain unresolved and (3) how can one evaluate the wider impacts of cash transfers on health and development?

#### Understanding mechanisms and pathways

##### Under what conditions and through what pathways do cash transfers influence HIV transmission?

Studies suggest that cash transfers work through many mechanisms and pathways. For example, was the impact of the Zomba trial driven by keeping girls in school, decreasing the number of sexual partners, changing the composition of sexual networks and/or decreasing the frequency of unprotected sex? Does the pathway vary by context (urban vs. rural setting) and/or by population (e.g. girls vs. boys at certain ages and income levels)? A better understanding of pathways and how they are affected by context would help to inform where and under what conditions cash transfers may be an effective part of HIV programme.

##### Are cash payments on their own empowering to women, or are complementary approaches needed?

Despite often being presented as an empowering intervention for women, the actual impacts of cash transfers on women's agency and household gender dynamics remain to be tested [26]. It is yet unclear whether cash transfers empower women and girls through the pathway of directly increasing their access to cash or of indirectly increasing their access to education or increased social networks. If one aim of cash transfers is to empower women, it is important that social and economic empowerment of women becomes an explicit objective of such programmes (with measurable indicators). Existing programmes may require complementary activities that explicitly address gender inequalities to be truly transformational and ensure equitable, sustained impacts [26].

#### Answering key operational and programmatic questions

##### To what extent is “conditionality” required for cash transfers to yield positive benefits in terms of HIV prevention?

Although much attention has been paid to the value of economic incentives as behavioural motivators, in practise, the HIV impacts shown in the Zomba trial were achieved in both the conditioned and non-conditioned arm of the trial. Given that incentive-based schemes are costly to maintain and difficult to implement and enforce well [27], are there settings or populations where it might be simpler and equally effective to provide cash transfers without conditions?

##### What size of transfer and degree of targeting are most effective and cost-effective?

Whilst targeting the most vulnerable maximizes potential impact, it can be costly. Operational research is needed to determine the trade-off of targeting only those most in need versus wider groups. The size of transfer needed to achieve the desired benefit is also open to investigation. Tiered payment structures in existing trials show non-linear responses, which suggests that optimizing transfer size is crucial. Cash transfers need to be expensive enough to generate an effect, but can run the risk of being more expensive than they need to be; thus, costing out such programmes is essential.

##### What perverse consequences might cash transfers or incentive schemes generate?

The potential for cash transfers to create perverse incentives or infringe on human rights is still being explored. For example, there is some evidence suggesting that CCTs might have resulted in an increase in fertility of 2–4 percentage points in Honduras because only pregnant women were eligible for the subsidy [28]. Similar effects on fertility were found in Panama, although this result was not sustained in all analyses [29]. Further evidence is needed to explore the potential for perverse incentives to yield negative consequences for HIV risk behaviour. Extra cash in the hands of men, for example, could lead to increased use of alcohol, drugs or commercial sex. Evaluations should be comprehensive and rigorous enough to capture potentially negative outcomes and provide insights into how such impacts could be minimized or avoided. Involving communities in programme design can further reduce these risks.

##### In what settings might cash transfer programmes be an important HIV prevention strategy for women?

Current evidence comes from settings with high HIV prevalence among women and adolescent girls in the general population. Are there other settings where such interventions could be important (e.g. in refugee camps, in migrant communities or among sex workers or intravenous drug-using women)? How do the benefits of cash transfers compare to those of other forms of economic or poverty reduction programme?

##### How can cash transfer programmes targeting HIV outcomes be sustainable and delivered at scale?

To be delivered at scale, it is likely that cash transfer programmes will need to be integrated into national social protection mechanisms. CCT programmes in Latin America were almost entirely initiated by governments as part of wider poverty reduction schemes. In contrast, many cash transfer programmes (conditional or unconditional) in sub-Saharan Africa have been delivered and financed outside of government at a relatively small scale [4]. In the long term, the most sustainable cash transfer models may be those where it is feasible to link cash transfers to existing social programmes offered by the government. In contexts where social welfare systems are being constructed, a golden opportunity exists to make such systems as HIV-sensitive as possible.

#### Evaluating wider impacts on health and development

##### What are the long-term impacts of CCTs?

Most cash transfers for HIV prevention have been evaluated over relatively short time frames. It is thus unclear how long any protective effect may last and whether protection is contingent on a continued incentive, either to the same cohorts as they age or to new cohorts as they become eligible. For example, an analysis of the RESPECT study found that 1 year after cash incentives to stay STI free were removed, the intervention impact had been sustained among men, but not among women. This suggests that for men, the provision of a short-term incentive led to longer term behaviour change, whilst for women, the behaviour was not sustained [30].

##### How can cash transfer programmes be optimized to benefit a wide range of health and development outcomes?

Cash transfers, like many structural interventions, have multiple potential impacts above and beyond HIV. Yet, a lot of research occurs in silos, missing opportunities to capture a wide array of downstream impacts. Where appropriate, HIV and health research should capture broader health, social, economic and environmental impacts of interventions. Similarly, upstream development interventions, in areas such as poverty reduction, should examine impacts on HIV and health. Multiple, cross-sector impacts are among the key benefits of structural interventions, and demonstrating such impacts is important for their consideration by policy makers.

Silos extend to policy and programmes as well. Although there is often substantial potential to achieve HIV and development synergies, these are not always sought. Integrating HIV and health components into existing cash transfer schemes would expand the impact of these programmes without creating expensive new projects whose costs are borne by HIV donors or the health sector alone. For cross-sector collaboration to work, specific mechanisms and structures are required to bring together relevant, multidisciplinary teams. HIV and health experts must work across silos to help social protection managers understand how to design, monitor and evaluate the programmes for HIV and health-related impacts.

##### How do the benefits of cash transfers compare to those of other social protection and/or economic empowerment programmes?

Cash transfers are but one mechanism in a range of social protection options that governments employ. All such programmes – from insurance to employment schemes – hold the potential either to benefit HIV-affected individuals and families or to promote risky behaviours. Of particular concern, for example, are the impacts of large public works programmes that put large sums of cash – delivered in set instalments – into men's hands. While alleviating poverty, such programmes might also increase risky behaviours by encouraging binge drinking and commercial sex as men take advantage of their newfound cash. Further research is needed to understand the potential negative consequences of such programmes. Programme designers and evaluators should consider the impacts of these other social protection mechanisms and income schemes on HIV-related outcomes.

##### How can the cost effectiveness of transfer programmes be assessed to encourage cost sharing across sectors?

Traditionally, the cost effectiveness of interventions is assessed against a single development outcome – the project's effectiveness at reducing unwanted pregnancy, for example, or reducing risk of contracting malaria. But, as noted in this article, the benefit of structural interventions is that they yield multiple benefits across various development outcomes. A challenge moving forward is how to capture these multiple benefits and encourage co-financing across sectors. If a cash transfer yields HIV and education benefits, for example, it may be appropriate for some programme costs to be shared between HIV dollars and the education sector. Even if a conditionality is not necessary for HIV benefits, but it is critical for education, the conditionality may still be useful to ensure maximum co-financing across sectors. Doing so might make the economic case for implementing the cash transfer scheme, perhaps at less cost to HIV-specific budgets. Recent analysis of the cost and benefits of the Zomba trial, for example, demonstrates that even a programme that is not cost effective when assessed by traditional means can be very cost effective when analyzed using co-financing models [31].

## Conclusions

Despite increasing evidence of their importance, cash transfers are by no means a simple solution to addressing HIV vulnerability. The history of the AIDS epidemic has repeatedly shown that even breakthrough prevention strategies, if used in isolation, cannot halt the epidemic. Rather, new approaches, if used in effective combinations and in the right contexts, can bend the epidemic's curve.

The emerging evidence suggests that cash transfers could be an important addition to HIV prevention efforts. Ongoing and future studies should unpack unresolved questions and collect additional evidence on the multiple impacts of transfers in different settings. To ensure sustained impact on HIV, cash transfer programmes need to be integrated with other sector programmes that address structural factors such as education and programming to promote gender equality [32].
